# Epicatechin inhibits inflammatory injury in preeclampsia extravillous trophoblasts

**DOI:** 10.1530/REP-24-0182

**Published:** 2025-08-05

**Authors:** Mengyongwei Li, Mian Liu, Jiaoqi Mei, Haofu Dai, Huiqin Chen, Qiuling Jie, Jingjing Mei, Xiaohui Yang, Jinyu Kang, Yanlin Ma, Wenli Mei

**Affiliations:** ^1^ Hainan Provincial Key Laboratory for Human Reproductive Medicine and Genetic Research, Hainan Provincial Clinical Research Center for Thalassemia, Department of Reproductive Medicine, The First Affiliated Hospital of Hainan Medical University, Hainan Medical University, Haikou, Hainan, China; ^2^ Key Laboratory of Reproductive Health Diseases Research and Translation (Hainan Medical University), Ministry of Education, Haikou, Hainan, China; ^3^ Haikou Key Laboratory for Preservation of Human Genetic Resource, The First Affiliated Hospital of Hainan Medical University, Hainan Medical University, Haikou, Hainan, China; ^4^ Department of Obstetrics and Gynecology, Reproductive Medicine, Nanfang Hospital, Southern Medical University, Guangzhou, Guangdong, China; ^5^ Key Laboratory of Natural Products Research and Development From Li Folk Medicine of Hainan Province, Institute of Tropical Bioscience and Biotechnology, Chinese Academy of Tropical Agricultural Sciences, Haikou, Hainan, China

**Keywords:** (−)-epicatechin, small-molecule compound, pyroptosis, inflammation, preeclampsia

## Abstract

**In brief:**

Preeclampsia is a severe pregnancy-related complication that can result in adverse maternal and fetal outcomes. Current therapeutic options for preeclampsia remain limited. This study demonstrates that epicatechin can inhibit pyroptosis in extravillous trophoblasts and block the activation of the NF-κB signaling pathway, thereby offering a novel therapeutic approach for the management of preeclampsia.

**Abstract:**

Preeclampsia (PE) is characterized as new-onset hypertension and proteinuria after 20 weeks of gestation, and affects 5–7% pregnant women globally. PE is associated with a systemic inflammatory status that is overly activated and contributes to dysregulated extravillous trophoblasts (EVTs) invasion and impaired spiral vessel remodeling. Recent studies showed that inhibition of systematic inflammatory response significantly ameliorates the PE-like symptoms, suggesting that anti-inflammation could be a potential PE treatment. However, few effective therapeutic strategies have been shown to control systemic inflammation in PE patients. In the current study, we investigated the protective effects of epicatechin (EC), a small molecule compound that exhibits excellent anti-inflammatory activity on HTR8/SVneo cells and EVTs stimulated with lipopolysaccharide (LPS). Our results revealed that EC pretreatment significantly improved cellular viability and attenuated the inflammatory response of EVTs in response to LPS stimulation. Mechanistically, we found that EC significantly blocked the activation of the LPS-induced pyroptosis pathway of classical pyrin domain protein 3, cleaved caspase 1 and cleaved gasdermin D (NLRP3/caspase-1/GSDMD) in LPS-treated EVTs and inhibited interleukin-1β (IL-1β) expression (a hallmark of pyroptosis) by suppressing the nuclear factor-κB (NF-κB) signaling. Our study demonstrates the protective effects of EC on LPS-stimulated inflammation and provides the direct evidence *in vitro* that EC may be a promising compound that mitigates the PE-associated systemic inflammation.

## Introduction

Preeclampsia (PE) is a severe pregnancy complication that is diagnosed by the development of hypertension and proteinuria after 20 weeks of pregnancy. PE affects 5–7% of pregnant women worldwide, and increases maternal and fetal mortality and morbidity. As part of the ‘large obstetric syndrome’, abnormal maternal inflammatory response is one of the key pathophysiological features of PE development (Erez *et al.* 2022), and hyperactivation of inflammation may affect the process of EVTs invasion, spiral artery remodeling. Growing evidence suggests that PE patients show an overactive inflammatory reaction, which is crucial to the development of PE (Deer *et al.* 2023).

Inflammation is an adaptive response to various stimuli and conditions, which plays an important role in a variety of physiological and pathological processes. It is generally believed that the inflammatory response within a certain range is beneficial to the human body; however, if it is not regulated, the inflammatory response will cause body damage (Liu *et al.* 2023). Women undergo a physical inflammatory response during embryonic implantation and in the first trimester of pregnancy, which is critical for successful implantation and placentation. However, numerous studies have demonstrated that the overly activated inflammatory status at the maternal–fetal interface contributes to the pathophysiology of PE (Kim *et al.* 2015, Romanowska-Próchnicka *et al.* 2021, Jin *et al.* 2022). Previous studies revealed a significant increase of pro-inflammatory cytokines such as IL-1β, TNF-α and IL-6 in PE patients and abrogating such over activated pro-inflammatory statues ameliorates the PE-like symptoms in rat models (Quan *et al.* 2021). These results suggest that anti-inflammation may be a potential therapeutic approach to treat PE patients.

A growing number of studies have shown that significant trophoblast pyroptosis has been found in patients with PE (Cheng *et al.* 2019, Nunes *et al.* 2021). Banerjee *et al.* (2021) reported that trophoblasts obtained from patients with early-onset PE showed significantly increased IL-1β and caspase-1 expression compared with those obtained from normal pregnant women and that exacerbated pyroptosis in trophoblasts contributed to the systemic inflammatory response in early-onset PE patients. Recent studies showed that PINK1-mediated mitophagy might play a protective role in PE by reducing ROS and EVTs pyroptosis, suggesting that pyroptosis in EVTs may comprise a potential therapeutic target for the treatment of PE patients (Sun *et al.* 2023). Thus, it is imperative to develop safe and effective therapies that can specifically target pyroptosis in the EVTs during PE in pregnancy.

To achieve high efficacy and avoid adverse effects, plant-derived natural compounds have been used as valuable sources of chemo-preventive agents in chronic inflammatory diseases such as psoriasis, pulmonary fibrosis, endometriosis, and PE (Park *et al.* 2020, Schimmel *et al.* 2020, Zhang *et al.* 2022). Among these plant-derived molecules, (−)-epicatechin (EC) is one of the potential therapeutic agents to treat chronic inflammatory disease (Bernatova 2018). Álvarez Cilleros *et al.* (2020) showed that EC protected against high glucose concentrations and lipopolysaccharide (LPS)-induced inflammation in renal proximal tubule cells through inhibiting NOX-4/p38 signaling. Thus, it is meaningful to investigate whether EC can serve as a potential therapeutic agent for PE by inhibiting EVTs pyroptosis.

In the current study, we revealed the protective role of EC in LPS-induced inflammatory response in cultured EVTs. EC significantly ameliorated LPS-induced inflammatory response of EVTs by inhibiting the pro-inflammatory cytokines. Mechanistically, EC inhibited LPS-induced pyroptotic activation in the EVTs, in part, by abrogating NOD-like receptor family pyrin domain-containing 3 (NLRP3) – apoptosis-associated speck-like protein containing a CARD (ASC) inflammasome formation, thus lessening the activation of the Caspase-1/GSDMD signaling pathway. In addition, EC reduced IL-1β expression by inhibiting the activation of the NF-κB signaling pathway. Thus, our study provides direct evidence *in vitro* to support the notion that EC may constitute a potential therapeutic agent to reduce over-activated inflammatory response in PE patients.

## Materials and methods

### Cell culture and treatments

HTR8/SVneo cells were purchased from the Chinese Academy of Science Cell Bank (China), grown in Dulbecco’s modified Eagle’s medium (DMEM)-high-glucose medium (Gibco, USA) supplemented with 10% FBS (Gibco, USA), and maintained at 37°C in a humidified incubator with 5% CO_2_. Then, in the follow-up experiment, HTR8/SVneo cells (3 × 10^5^) were incubated in a 6 cm petri dish at 37°C for 12 h until the cell adhesion growth was observed. Among them, the positive control cells were treated with vehicle, i.e. DMSO with the final concentration of 0.1%, and the negative control cells were in complete media. The LPS group was treated with 200 ng/mL LPS (Sigma, serotype 0127:B8, GER) for 24 h. The EC group was pretreated with 1 and 2 μM EC for 24 h, and then treated with 200 ng/mL LPS for 24 h. During the whole experiment, the concentration of DMSO in the medium was less than 0.1%.

### Isolation and cell culture of human primary EVTs

Abortion tissues of 6–8 weeks of early pregnancy were collected, and human primary EVTs were isolated according to previously reported method (Takao *et al.* 2011, Mei *et al.* 2024). Villi were rinsed three times with PBS, and then cut into small pieces and digested using 0.25% trypsin and 0.1 mg/mL DNase I at 37°C for 15 min. Cell suspension was collected and centrifuged at 18 ***g*** for 3 min. The above procedure was repeated another time. Cells were collected and then placed on ice. The cellular pellets were resuspended and layered on the top of a preformed Percoll gradient (65, 55, 50, 45, 35, 30, and 25%) and centrifuged at 25 ***g*** for 20 min at 4°C. The EVTs were then collected in the 30 and 50% Percoll solution layers, washed with PBS, plated on a Matrigel-coated culture surface, and cultured in DMEM/F12 (Gibco, USA) with 10% FBS (Gibco, USA) at 37°C in a humidified incubator with 5% CO_2_. All procedures were approved by the Hainan Provincial People’s Hospital and the First Affiliated Hospital of Hainan Medical University, and all subjects gave written consent for the collection of tissue samples.

### Assessment of cellular viability

An MTT cell proliferation and cytotoxicity detection kit (KeyGEN, China) was adopted to measure cellular viability. HTR8/SVneo cells (5 × 10^3^) and EVTs (5 × 10^3^) were inoculated in 96-well plates and incubated at 37°C and 5% CO_2_ for 12 h until cells were attached to the wall. They were subsequently treated with EC (at a final concentration gradient of 0–4 μM and 0–30 μM) for 24 h. OD values (optical density) were measured at 490 nm with an enzyme-labeled instrument after culture at 37°C for 4 h with MTT solution.

### Cell proliferation assay

HTR8/SVneo cells (1 × 10^4^) were seeded in 24-well plates for 24, 48, 72, and 96 h at 37°C and 5% CO_2_ and counted separately until the end of 96 h for statistical analysis. Cell proliferation was determined using a Cell-Light EdU Apollo567 Kit (RiboBio, China), according to the manufacturer’s instructions. After incubation with 50 μM EdU for 2 h, cells were fixed with 4% paraformaldehyde (Biosharp, China) and sealed with Apollo reaction cocktail and Hoechst 33342. All images were acquired with a fluorescence microscope (Olympus, Japan).

### RNA isolation and quantitative real-time PCR

Total RNA was extracted using TRIzol reagent (Invitrogen Life Technologies, USA) based on standard procedures. cDNA was prepared with the PrimeScript® RT reagent Kit (Takara, Japan) according to the manufacturer’s instructions. We quantified the expression levels of gene mRNA using an SYBR^®^ Premix Ex Taq TM II kit (Takara, Japan). Relative expression levels were determined by normalizing the expression level of each target to glyceraldehyde 3-phosphate dehydrogenase (GAPDH), and relative fold-changes in mRNA were determined using the 2^−ΔΔCt^ method. Samples were run in triplicate. All primer sequences are shown in Table 1.

**Table 1:** Primers used in RT-qPCR.

Gene	Accession numbers	Primer sequences	
		Forward	Reverse
*IL-6*	NM_000600	ACT​CAC​CTC​TTC​AGA​ACG​AAT​TG	CCA​TCT​TTG​GAA​GGT​TCA​GGT​TG
*IL-8*	NM_001310420	CAC​ACT​GCG​CCA​ACA​CAG​AAA	GCA​CCC​AGT​TTT​CCT​TGG​GG
*TNF-α*	NM_001124357	GAG​GCC​AAG​CCC​TGG​TAT​G	CGG​GCC​GAT​TGA​TCT​CAG​C
*IL-1β*	NM_008361	CCA​CAG​ACC​TTC​CAG​GAG​AA	GTG​ATC​GTA​CAG​GTG​CAT​CG
*IL-18*	NM_001243211	TGC​ATC​AAC​TTT​GTG​GCA​AT	ATA​GAG​GCC​GAT​TTC​CTT​GG

### Western blotting

Total proteins were purified from cells or tissues, denatured by boiling, separated by sodium dodecyl sulfate polyacrylamide gel electrophoresis (SDS-PAGE, Beyotime, China) and transferred onto polyvinylidene fluoride (PVDF, Beyotime, China) membranes. After blocking with 3% bovine serum albumin (BSA, Beyotime, China) in Tris-buffered saline containing 0.1% Tween-20 (TBST), the membrane was combined with anti-phosphorylated P65 antibody (1:1,000, 3033T, CST), anti-IL-1β precursor and mature antibody (1: 1,000,12703, CST), anti-NLRP3 antibody (1:1,000, NBP2-12446, Novus Biologicals, USA), anti-caspase-1 antibody (1:1,000, ab179515, Abcam, UK), and anti-GSDMD-N-terminal antibody (1:1,000, ab210070, Abcam) and incubated overnight. Then, the cells were incubated with HRP-conjugated anti-rabbit/mouse secondary antibodies (1:10,000) for 1 h at room temperature. Finally, the specific protein bands were visualized with a chemiluminescent substrate kit (Millipore, USA), and images were captured using a Tanon 4600SF instrument (Tanon, China). The densitometric analyses were performed using the ImageJ^®^ software (NIH, USA), and results were analyzed using GAPDH (1: 2,000, ab8245, Abcam), histone H3 (1:2,000, ab986936, Abcam) and NF-κB P65 total protein (1:1,000, 8242T, CST) as internal controls.

### Enzyme-linked immunosorbent assay (ELISA) of cytokines

According to the manufacturer’s instructions, the cytokine levels in culture medium were quantified using TNF-α (Cusabio, China), IL-6 (Cusabio, China), IL-1β (Cusabio, China), IL-18 (Cusabio, China) and IFN-γ (Sangon, China) kits.

### Immunofluorescence

Cells grown on cover slides were fixed with methanol and acetone (1:1) at −20°C for 20 min, permeabilized with 0.5% Triton X-100 for 20 min, blocked with 5% BSA (Beyotime, China) in phosphate-buffered saline (TBST) containing 0.1% Tween-20 for 1 h, and then incubated overnight with anti-NLRP3 antibodies (1:200, NBP2-12446, NovusBio) and anti-ASC antibodies (1:200, ab283684, Abcam). The slides were incubated with Alexa Fluor® 488 IgG anti-mouse (1:1,000, ab150117, Abcam) and Alexa Fluor® 594 IgG anti-rabbit (1:1,000, ab150080, Abcam) antibodies in the dark for 1 h. The nuclei were stained with 4′,6-diamidino-2-phenylindole (DAPI). Fluorescence images were acquired using a confocal microscope (Olympus Fluoview FV3000, Japan).

### Cellular fractionation

Cell nuclei and cytoplasm were separated using a membrane, nuclear, and cytoplasmic protein extraction kit (Sangon, China), according to the manufacturer’s instructions. Briefly, culture medium was removed, and cells were washed with ice-cold PBS, scraped off in PBS, and centrifuged at 16 ***g*** for 5 min. Cells were then suspended in hypotonic lysis buffer containing protease and phosphatase inhibitors and incubated on ice for 15 min. The homogenate was centrifuged at 26 ***g*** for 30 s to separate the nuclear extracts (pellet) from the cytoplasmic extracts (supernatant). The isolated nuclear protein extracts were then resuspended and incubated in ice-cold hypertonic lysis buffer for 40 min, followed by centrifugation at 26 ***g*** for 10 min at 4°C, and the supernatant containing the nuclear proteins was collected and assayed.

### Statistical analysis

All statistical analyses were conducted using the SPSS software package (version 26.0, SPSS, Inc., USA) and Prism 8.0 software (GraphPad, USA). Data are presented as the means ± standard deviation (SD) of at least three independent experiments. The *t*-test was used to analyze the significant differences of data between two groups, and the one-way ANOVA followed by a post-hoc Tukey’s test was used for data comparisons among multiple groups. The results were considered to be statistically significant at the level of *P* < 0.05.

## Results

### EC protects HTR8/SVneo and EVTs from LPS-induced injury

**Figure 1:**
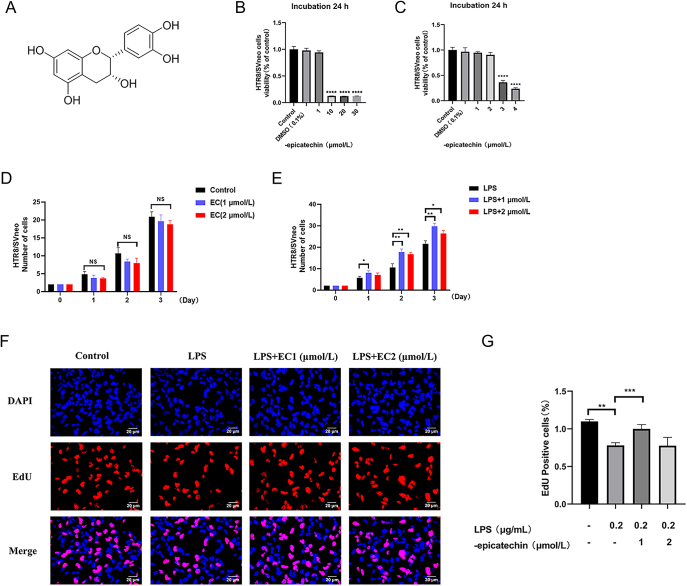
EC protects HTR8/SVneo from LPS-induced injury. (A) Structural formula of epicatechin. (B) Epicatechin concentrations greater than or equal to 10 μM significantly decreased cellular survival rate. (C) Epicatechin concentrations above 3 μM significantly decreased cell survival rate. Concentrations less than or equal to 2 μM exerted no discernible effect on cellular survival rate. (D) Statistical analysis of effects of EC on HTR8/SVneo cells in the control group. (E) Statistical analysis of effects of EC on HTR8/SVneo cells in the inflammatory group. (F) Proliferation experiment, with DAPI as an internal control. (G) Statistical analysis of the proliferation experiments (scale = 50 μm). Data are expressed as the mean ± SEM (*n* = 5 for each group). *P < 0.05, **P < 0.01, ***P < 0.001, ****P < 0.0001 compared to the control group. DAPI, 4′,6-diamidino-2-phenylindole; EdU, 5-ethynyl-2′-deoxyuridine; control, normal untreated HTR8/SVneo cells; LPS, HTR8/SVneo cells treated with LPS.

Toxicity tests were performed to determine the safe concentration of EC. After 24 h incubation with cells, EC concentrations of 1 and 2 μM showed no significant effects on HTR8/SVneo and primary EVTs (*P* > 0.05) (Fig. 1B and C, Supplementary Material S1 (see the section on Supplementary materials given at the end of the article), Fig. 1). Thus, these two concentrations were then selected for subsequent experiments throughout this study. We observed no significant difference in the number of EC-treated HTR8/SVneo cells compared with the control group 3 days before EC treatment at 1 and 2 μM (*P* > 0.05) (Fig. 1D). After stimulation with 200 ng/mL LPS, the number of HTR8/SVneo cells in the EC treatment group was significantly higher from that in the LPS group, with 1 μM EC on reversing LPS-induced cell damage being the most significant (LPS and LPS + 1 μM: *P* < 0.01 and LPS and LPS + 2 μM: *P* < 0.05) (Fig. 1E).

The number of EdU of HTR8/SVneo cells was reduced after stimulation with LPS compared to the control group, which implies a decrease in proliferative capacity; in contrast, the drug group showed an increase in the number of EdU cells after 1 μM EC treatment compared to the LPS group, suggesting an increased proliferative capacity, while there was no change after 2 μM EC treatment (Fig. 1F and G) (control and LPS: *P* = 0.0003 and LPS and LPS + 1 μM: *P* = 0.005). These results demonstrated that EC exerts a protective effect against LPS-induced cell damage and that 1 μM EC was superior to results with 2 μM.

### Effects of EC on the level of pro-inflammatory cytokines from HTR8/SVneo and EVTs treated with LPS

**Figure 2:**
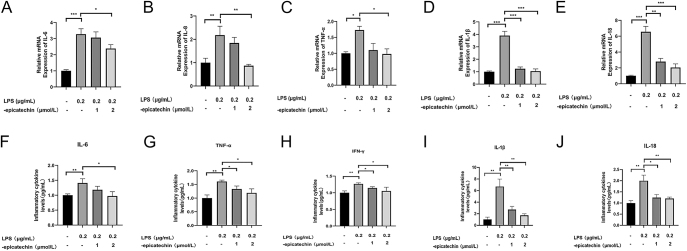
Anti-inflammatory activity of EC. (A, B, C, D, E) Expression of IL-6, IL-8, TNF-α, IL-1β and IL-18 mRNA in HTR8/SVneo cells detected by real-time PCR. (F, G, H, I, J) Expression of IL-6, TNF-α, IFN-γ, IL-1β and IL-18 protein levels in the supernatant of HTR8/SVneo cell culture medium detected by ELISA. *P < 0.05, **P < 0.01, ***P < 0.001, compared with the control group.

To investigate whether varying concentrations of EC exhibit differential anti-inflammatory activities, HTR8/SVneo and EVTs cells were preincubated with different concentrations of EC for 24 h, followed by induction of an inflammatory response using LPS. Subsequently, RT-qPCR was employed to quantify the mRNA expression levels of common inflammatory markers including IL-6, IL-8, TNF-α, IL-1β, and IL-18 across all groups. The results showed that the mRNA levels of inflammatory factors were all significantly increased after LPS stimulation compared with the control group, and the mRNA levels of IL-6, IL-8 and TNF-α were downregulated after pretreatment with EC (Fig. 2A, B, C) (IL-6: LPS and LPS + 2 μM: *P* = 0.0203, IL-8: LPS and LPS + 2 μM: *P* = 0.0043, and TNF-α: LPS and LPS + 2 μM: *P* = 0.034). Interestingly, we found that EC significantly inhibited the mRNA levels of cellular pyroptosis marker proteins, such as IL-1β and IL-18 (Fig. 2D and E) (IL-1β: LPS and LPS + 1 μM: *P* = 0.0002, LPS and LPS + 2 μM: *P* = 0.0002, IL-18: LPS and LPS + 1 μM: *P* = 0.0012, and LPS and LPS + 2 μM: *P* = 0.0007). HLA-G is a special type I major histocompatibility complex molecule mainly expressed on EVTs. As a biomarker of EVTs, we successfully identified the isolated cells by immunofluorescence detection. After the same experiments, we found similar evidence in primary EVTs, and the RT-qPCR results agreed with the results of the analysis of HTR8/SVneo cells (S1, Fig. 2 A, B, C, D, F).

Subsequently, we used ELISA assay to detect changes in the protein levels of inflammatory factors in the supernatants of HTR8/SVneo and primary EVTs culture medium. Consistent with the results of RT-qPCR analysis, EC pretreatment was able to reduce the protein expression levels of inflammatory factors (Fig. 2F, G, H) (IL-6: LPS and LPS + 2 μM: *P* = 0.0217, TNF-α: LPS and LPS + 2 μM: *P* = 0.0106, and IFN-γ: LPS and LPS + 2 μM: *P* = 0.0365); at the same time, we likewise observed that EC could significantly reduce the protein expression levels of IL-1β and IL-18 (Fig. 2I and J) (IL-1β: LPS and LPS + 1 μM: *P* = 0.008, LPS and LPS + 2 μM: *P* = 0.0028, IL-18: LPS and LPS + 1 μM: *P* = 0.0103, and LPS and LPS + 2 μM: *P* = 0.0061). Consistent evidence was also found in EVTs (S1, Fig. 2 G, H, I, J, K). These results suggest that EC can inhibit the expression of LPS-induced inflammatory cytokines in a dose-dependent manner, that is, 2 μM EC has a better anti-inflammatory effect than 1 μM EC. At the same time, EC is highly likely to protect EVTs by inhibiting pyroptosis, thereby reducing inflammatory damage.

### EC inhibits the release of IL-1β through blockade of the NF-κB signaling pathway

**Figure 3:**
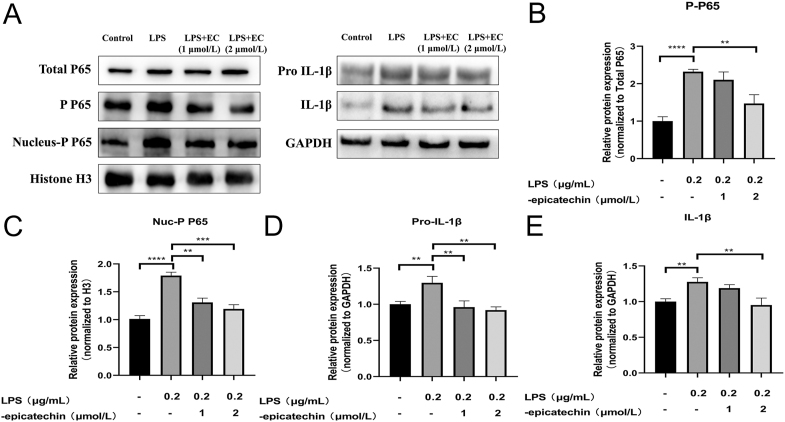
Regulation of NF-κB signaling by EC in HTR8/SVneo cells. (A) Representative images of total P65 subunit and phosphorylation of the NF-κB signaling pathway, intracellular P65 subunit, pro-IL-1β, and IL-1β by Western blotting analysis. (B) Quantitative analysis of P65 subunit phosphorylation and total protein of P65 was utilized as the internal control. (C) Quantitative analysis of the P65 subunit in the nucleus and histone H3 was utilized as the internal control. (D) Quantitative analysis of IL-1β precursors and GAPDH was utilized as the internal control. (E) Quantitative analysis of IL-1β and GAPDH was utilized as the internal control. Data are expressed as the mean ± SEM (*n* = 3 for each group). **P < 0.01, ***P < 0.001, ****P < 0.0001, compared to the control group.

In order to explore the specific mechanism of EC’s anti-inflammatory activity, we selected the classical inflammatory signaling pathway NF-κB for experiments. We observed enhanced phosphorylation of the P65 subunit in the NF-κB signaling pathway in HTR8/SVneo cells after LPS stimulation compared to the control group (Fig. 3A and B) (*P* < 0.0001) and plasma nuclear isolation and detection of the P65 subunit in the nucleus of HTR8/SVneo cells revealed an increase in the number of phosphorylated P65 subunits in the nucleus (Fig. 3A and C) (*P* < 0.0001). The phosphorylation level of NF-κB pathway P65 subunit and the amount of phosphorylated P65 subunit in the nucleus were reduced in EC-treated HTR8/SVneo cells compared with the LPS group (Fig. 3A, B, C) (P-P65: LPS and LPS + 2 μM: *P* = 0.0038 and Nuc-P P65: LPS and LPS + 2 μM: *P* = 0.0005). Thereafter, detecting the NF-κB pathway downstream gene IL-1β and its precursor, we found that the levels of pro-IL-1β and IL-1β proteins were elevated in the LPS group, and the proteins were decreased after EC pretreatment (Fig. 3A, D, E) (pro-IL-1β: LPS and LPS + 2 μM: *P* = 0.0038 and IL-1β: LPS and LPS + 2 μM: *P* = 0.008). Performing the same experiment in EVTs further validates our results (S1, Fig. 3). These results suggest that EC inhibits the phosphorylation of the P65 subunit and prevents its entry into the nucleus, ultimately inhibiting the activation of the NF-κB signaling pathway.

### EC attenuates LPS-induced inflammasome expression in HTR8/SVneo and EVTs inhibit the NLRP3/caspase-1/GSDMD signaling pathway

**Figure 4:**
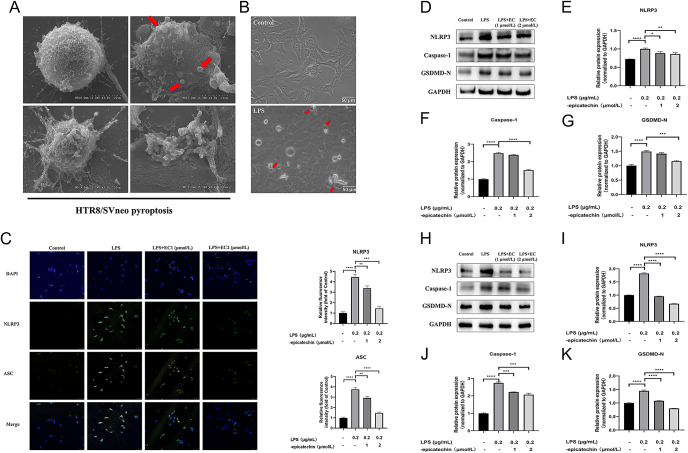
Effects of EC on cellular pyroptosis. (A) Morphological changes of HTR8/SVneo cells after different times of LPS stimulation observed under transmission electron microscope (from left to right, normal morphology cells, cell membrane swelling, cell membrane cleavage, cell pyroptosis) (B) Morphological changes of HTR8/SVneo cells were observed under an optical microscope (red arrows indicate cells that are already in pyroptosis). (C) The assembly and quantitative analysis of inflammatory bodies in HTR8/SVneo cells were observed by immunofluorescence assay, and DAPI was used as the internal control for nuclear staining. (D) Representative images of pyroptosis-related proteins NLRP3, caspase-1, and GSDMD-N terminus analyzed by Western blotting in HTR8/SVneo cells. (E) Quantitative analysis of NLRP3. (F) Quantitative analysis of caspase-1. (G) Quantitative analysis of GSDMD-N. (H) Representative images of pyroptosis-related proteins NLRP3, caspase-1, and GSDMD-N terminus as analyzed by Western blotting in human primary chorionic trophoblast cells. (I) Quantitative analysis of NLRP3. (J) Quantitative analysis of caspase-1. (K) Quantitative analysis of GSDMD-N (scale = 50 um). GAPDH served as the internal control in all analyses of Western blotting results. Data are expressed as the mean ± SEM (*n* = 3 for each group). *P < 0.05, **P < 0.01, ***P < 0.001, ****P < 0.0001 compared to the control group. DAPI, 4′,6-diamidino-2-phenylindole; NLRP3, NOD-like receptor family pyrin domain-containing 3 fluorescence image; ASC, a fluorescence image of apoptosis-associated speck-like protein containing a CARD.

In prior experiments, we observed the anti-inflammatory activity of EC by selecting several common inflammatory factors. Our findings revealed that EC could markedly suppress the levels of pro-inflammatory cytokines IL-1β and IL-18, which warranted further investigation. To visualize the cellular pyroptosis, we first photographed HTR8/SVneo cells after LPS stimulation at different times under transmission electron microscope (Fig. 4A), and the cells showed typical features of pyroptosis, such as swelling and crumpling, under LPS stimulation. Next, we also obtained images under an optical microscope that were consistent with transmission electron microscopy (Fig. 4B).

In immunofluorescence experiments, we observed increased expression levels of NLRP3 and ASC in HTR8/SVneo cells after LPS stimulation compared with the control group as revealed by the inflammasome-specific antibodies, which were reduced by EC treatment (Fig. 4C). Consistent with the above observations, Western blot assays also showed that NLRP3, caspase-1, and GSDMD were upregulated in the LPS group but were reduced after EC treatment (Fig. 4D, E, F, G) (NLRP3: LPS and LPS + 1 μM: *P* = 0.0158, LPS and LPS + 2 μM: *P* = 0.006, caspase-1: LPS and LPS + 2 μM: *P* < 0.0001, and GSDMD-N: LPS and LPS + 2 μM: *P* = 0.0001). Similar observations were obtained from human primary EVTs (Fig. 4H, I, J, K) (NLRP3: *P* < 0.0001, caspase-1: LPS and LPS + 1 μM: *P* = 0.0001, LPS and LPS + 2 μM: *P* = 0.0004, and GSDMD-N: *P* < 0.0001).

These results suggested that EC inhibits the expression of the LPS-induced inflammasome in EVTs, thereby inhibiting the activation of NLRP3/caspase-1/GSDMD signal transduction, a classical pyroptosis pathway.

## Discussion

An excessive inflammatory response at the maternal–fetal interface results in serious pregnancy-related complications, such as PE or fetal growth restriction, which impose long-term negative effects on both women and their fetuses (Burton & Jauniaux 2018, Brosens *et al.* 2019). Recent studies have suggested that women with preterm PE possessed a significantly increased risk of cardiovascular complications associated with persistent overactivation of their inflammatory status (Erez *et al.* 2022). Thus, it is important to ameliorate the overactivated inflammation characteristic of PE patients.

A large amount of evidence reveals that small-molecule compounds extracted from plants show anti-inflammatory activities that could provide potential treatment for a variety of inflammation-related diseases with high efficacy and few adverse effects (Xuan *et al.* 2016, Mu *et al.* 2020). There have been numerous *in vivo* studies of EC, which highlight the safety of EC (Takagaki & Nanjo 2013, Chun *et al.* 2022). Although many studies have demonstrated the protective role of EC in inflammatory diseases by suppressing the inflammatory response (Cremonini *et al.* 2020, Álvarez Cilleros *et al.* 2020), the effect of EC on inflammatory injury in extracellular trophoblasts is unknown. A recent study by Xu showed that epigallocatechin gallate can improve PE-like symptoms in mice, demonstrating that it is feasible to improve PE by inhibiting inflammation, and raising a question that, as a precursor of epigallocatechin gallate, whether EC can also improve the clinical symptoms of PE by inhibiting inflammation (Xu *et al.* 2024). In this study, we focused on the anti-inflammatory effect of EC on LPS-induced HTR8/SVneo and EVTs *in vitro* and discussed whether EC could be a potential therapeutic agent with good therapeutic efficacy in patients with preeclampsia. We proved it here for the first time that EC increased viability and attenuated the pro-inflammatory conditions in HTR8/SVneo and EVTs induced by LPS. Our results revealed that EC pretreatment significantly decreased the expression of pro-inflammatory mediators such as TNF-α and IL-1β (classical hallmarks of pyroptosis), suggesting that EC protected HTR8/SVneo and EVTs from LPS-induced inflammation by suppressing activation, and alleviating the progression of PE disease.

Pyroptosis is an important inflammatory programmed necrosis that is characterized by NLRP3 inflammasome-promoted and caspase-1-dependent plasma membrane rupture. Pyroptosis releases damage-associated molecular patterns (DAMPs) and cytokines such as IL-1β into the extracellular environment, causing systemic inflammatory overactivation (Bergsbaken *et al.* 2009). A recent study has shown that the overactivation of pyroptosis in EVTs contributes to the development of PE and that the inhibition of the activation of EVTs pyroptosis significantly ameliorates PE-like symptoms (Kohli *et al.* 2016). This suggests that pyroptosis is likely to be a very critical link in the progression of PE patients. Our results showed that EC treatment attenuated the LPS-induced pyroptotic activation of HTR8/SVneo and EVTs by inhibiting the NLRP3/caspase-1/GSDMD classical pyroptosis signaling pathway and reducing pro-inflammatory cytokine IL-1β transcription and release. This shows the huge clinical therapeutic potential of EC. Therefore, we believe that EC is likely to alleviate the overactivation of systemic inflammation in PE patients and improve the clinical outcome of PE patients by inhibiting pyroptosis.

At the same time, we found a noteworthy phenomenon in the experiment, that is, EC can also inhibit the release of the downstream factor IL-1β in the NF-κB signaling pathway. As a classic inflammatory signaling pathway, NF-κB is involved in the progression of a variety of inflammatory diseases.(Yu *et al.* 2020). In our experiments, we found that EC inhibits the activation of the NF-κB signaling pathway by inhibiting the phosphorylation of the NF-κB P65 subunit and blocking its translocation to the nucleus. These results suggest that EC can not only significantly inhibit pyroptosis and relieve systemic inflammatory symptoms in PE patients, but also alleviate the overactivated inflammatory response in PE patients by inhibiting other inflammatory pathways. It is believed that with the continuous deepening of research, there will be more and more evidence showing the huge role of EC in inflammatory diseases in the future.

Nevertheless, we must admit that this study still has some limitations. First of all, we noted that although lower concentrations of EC had no significant effect on the activity of HTR8/SVneo cells and EVTs, greater cytotoxicity began when the concentration was greater than 3 μM, and more rigorous experiments were needed to further explore the safety of EC. Next, we note that the anti-inflammatory effect of EC is dose-dependent, that is, high doses of EC show better anti-inflammatory activity, so how we can maintain low cytotoxicity of EC and still have high anti-inflammatory activity is a big challenge for us in subsequent studies. Finally, we believe that although EC shows an excellent inhibition of pyroptosis, it is still unable to eliminate all the effects of LPS. Whether EC is only aimed at alleviating the damage caused by the inflammatory symptom of pyroptosis, and whether it also has an inhibitory effect on other inflammatory injuries, we will use a more rigorous and scientific method to further study.

In conclusion, our study provides a new direction for the clinical treatment of PE patients. EC can improve the pregnancy status of PE patients by inhibiting the overactivation of inflammation. In terms of mechanism, EC mainly inhibits pyroptosis by inhibiting the assembly of inflammatory bodies, and at the same time, EC can inhibit the activation of NF-κB signaling pathway by inhibiting the entry of P65 subunits into the nucleus. Nevertheless, more in-depth and comprehensive studies are needed in the future to demonstrate the great potential of EC in treating PE patients.

## Supplementary materials



## Declaration of interest

The authors declare that there is no conflict of interest that could be perceived as prejudicing the impartiality of the work reported.

## Funding

Supported by the Major Science and Technology project of Hainan Provincehttps://doi.org/10.13039/501100013072 (ZDKJ2021037), the National Natural Science Foundation of Chinahttps://doi.org/10.13039/501100001809 (82201874, 81960283), the Natural Science Foundation of Hainan Provincehttps://doi.org/10.13039/501100004761 (822MS175), the Key Research and Development Project of Hainan Provincehttps://doi.org/10.13039/501100013142 (ZDYF2022SHFZ311), the Hainan Provincial Science and Technology Program for Clinical Medical Research Center (LCYX202203, LCYX202102, LCYX202301), specific research fund of The Innovation Platform for Academicians of Hainan Province and Hainan Province Clinical Medical Center.

## Author contribution statement

LMYW, MYL and LM were involved in experimental design and article revision. MJQ, KJY, YXH, MJJ and JQL helped in data analysis, literature review and experimental operation. DHF, CHQ and MWL helped in experimental design and paper writing. All authors contributed to its critical review and agreed on the final version.

## Data availability

The datasets used and/or analyzed during the current study are available from the corresponding author on reasonable request.
