# Supplementary material for: Epicatechin inhibits inflammatory injury in preeclampsia extravillous trophoblasts
Source: Reproduction. 2025 Aug 5;170(2):e240182. doi: 10.1530/REP-24-0182 (PMC12329818; doi:10.1530/REP-24-0182)
Supplement: Supplementary file 1 [file supplementary_materials.pdf]

supplementary material:

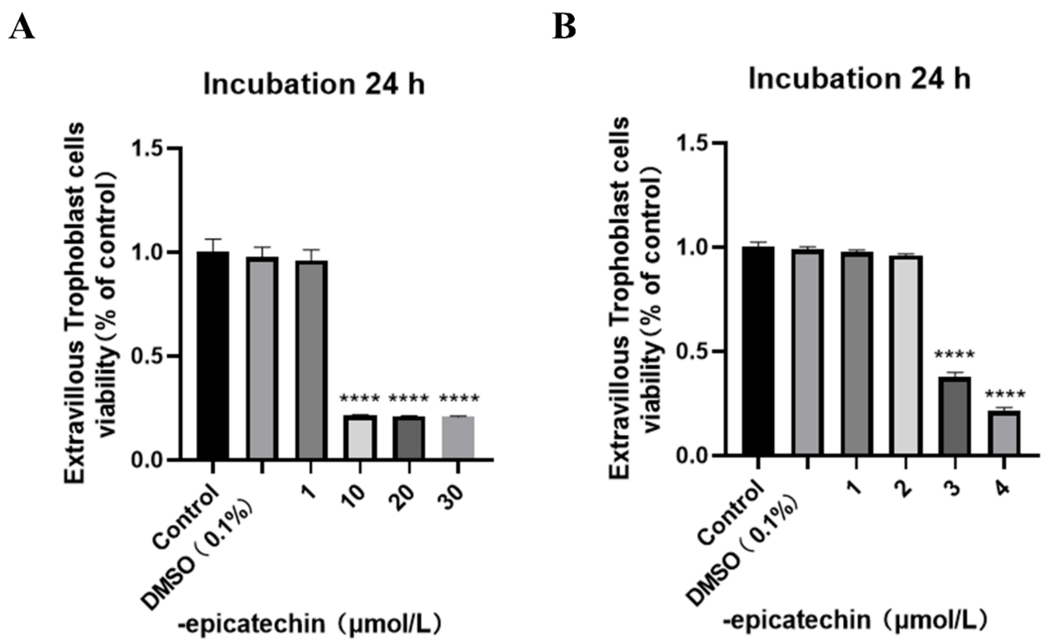

Figure 1. EC protects EVTs from LPS-induced injury. (A) Epicatechin concentrations greater than or equal to 10 μM significantly decreased cellular survival rate. (B) Epicatechin concentrations above 3 μM significantly decreased cell survival rate. Concentrations less than or equal to 2 μM exerted no discernible effect on cellular survival rate.

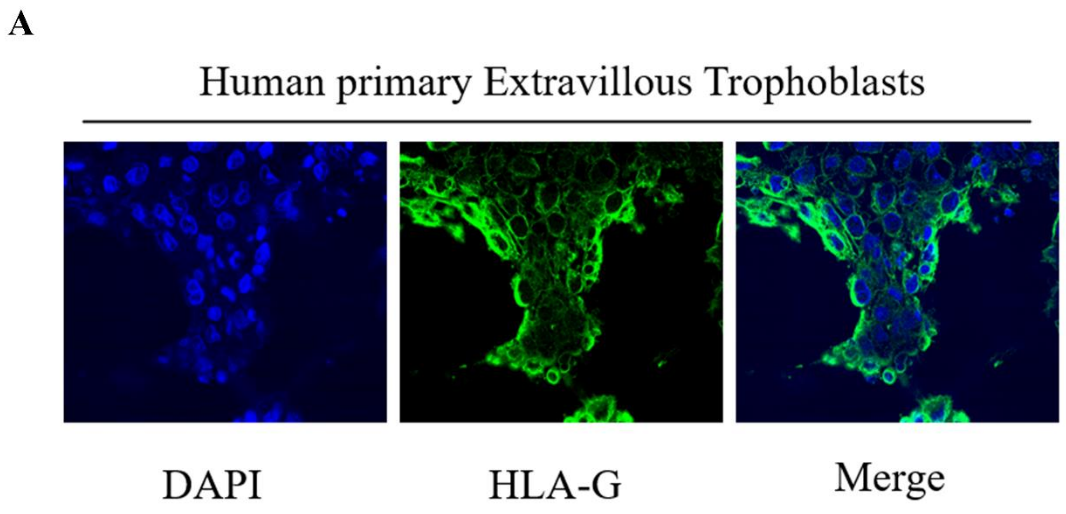

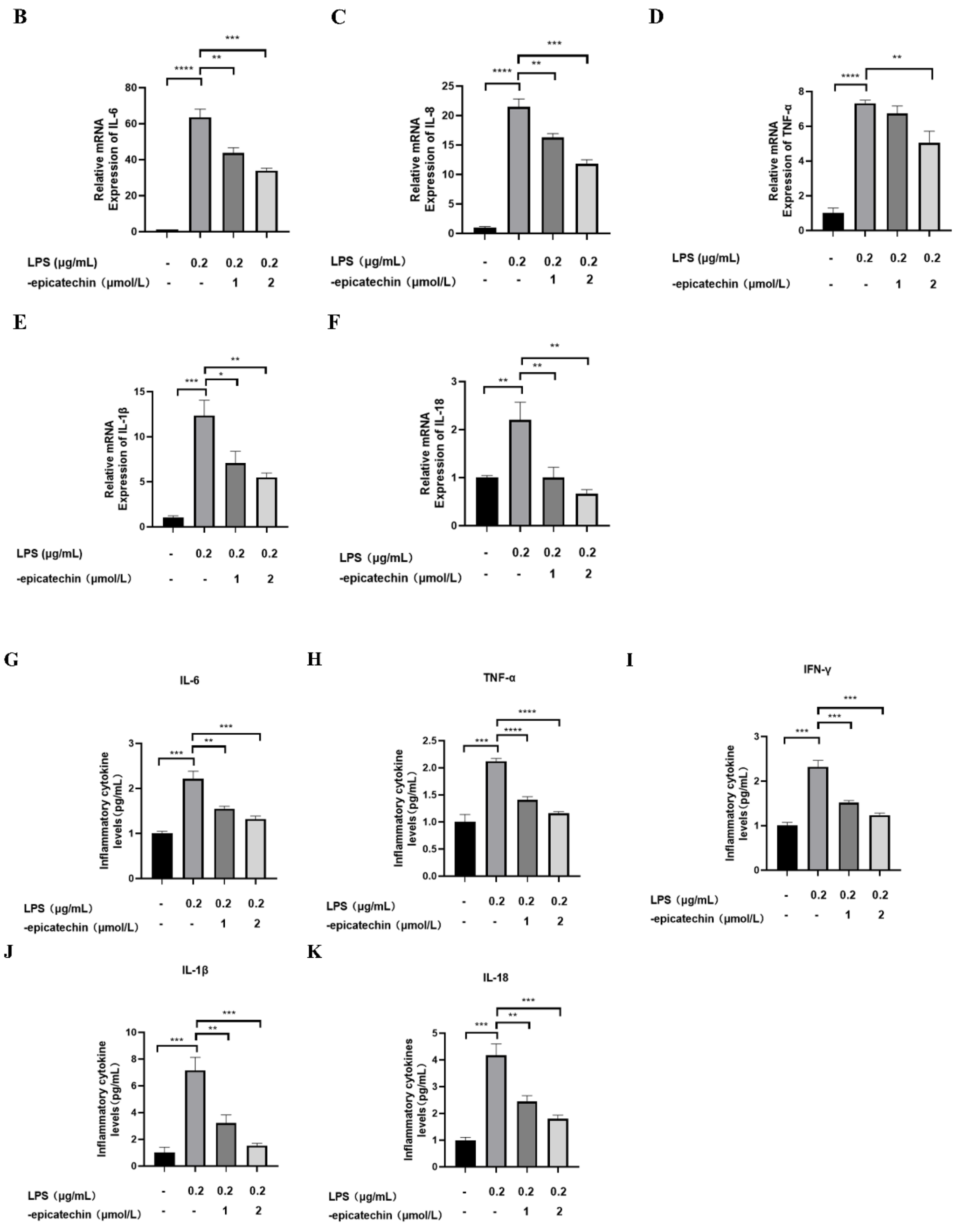

Figure 2. Anti-inflammatory activity of EC against LPS-induced EVTs. (A) Immunofluorescence assay to identify isolated EVTs. (B-F) Expression of IL-6, IL-8, TNF-α, IL-1β and IL-18 mRNA in HTR8/SVneo cells detected by real-time PCR. (G-K) Expression of IL-6, TNF-α, IFN-γ, IL-1β and IL-18 protein levels in the supernatant of HTR8/SVneo cell culture medium detected by ELISA. **P*<0.05, ***P*<0.01, ****P*< 0.001, compared with the control group. HLA-G, Human Leukocyte Antigen G; DAPI, 4’,6-diamidino-2-phenylindole

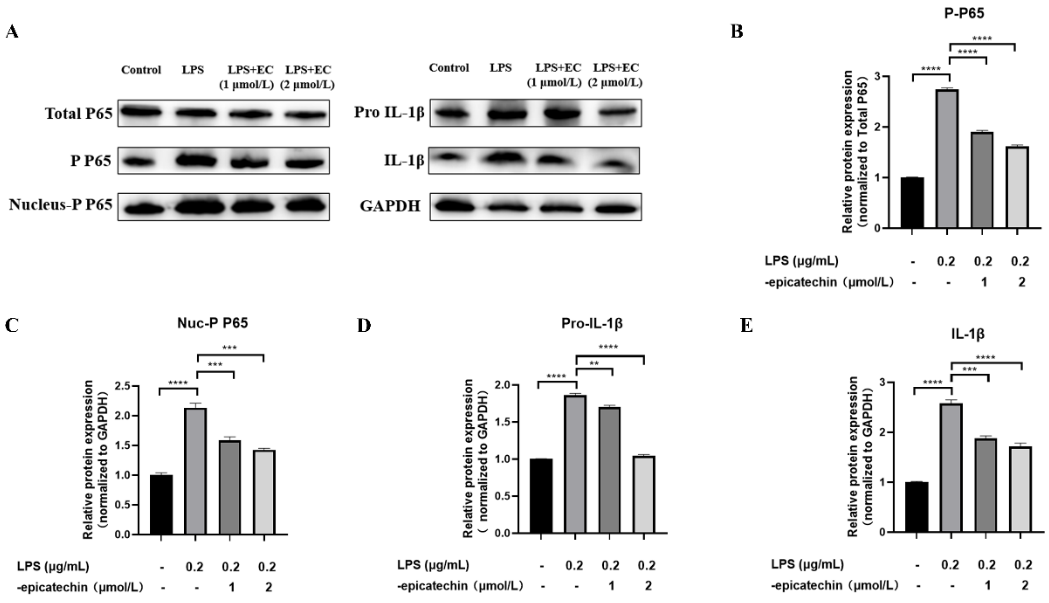

Figure 3. Regulation of NF-κB signaling by EC in EVTs. (A) Representative images of total P65 subunit and phosphorylation of the NF-κB signaling pathway, intracellular P65 subunit, pro-IL-1β, and IL-1β by Western blotting analysis. (B) Quantitative analysis of P65 subunit phosphorylation and total protein of P65 was utilized as the internal control. (C) Quantitative analysis of the P65 subunit in the nucleus and Histone H3 was utilized as the internal control. (D) Quantitative analysis of IL-1β precursors and GAPDH was utilized as the internal control. (E) Quantitative analysis of IL-1β and GAPDH was utilized as the internal control. Data are expressed as mean ± SEM (n = 3 for each group). $^{**}P<0.01$, $^{***}p < 0.001$, $^{****}p < 0.0001$ compared to the control group.
